# What Roles Do Probiotics Play in the Eradication of *Helicobacter pylori*? Current Knowledge and Ongoing Research

**DOI:** 10.1155/2018/9379480

**Published:** 2018-10-16

**Authors:** Han-Yi Song, Long Zhou, Dong-yan Liu, Xin-Jie Yao, Yan Li

**Affiliations:** ^1^Department of Gastroenterology, Shengjing Hospital of China Medical University, Shenyang, China; ^2^Department of Orthopedics, Shengjing Hospital of China Medical University, Shenyang, China; ^3^Medical Research Center, Shengjing Hospital of China Medical University, Shenyang, China

## Abstract

With the rising global prevalence of antibiotic resistance, the eradication rate of *Helicobacter pylori* (HP) is continuing to decrease. Probiotics are beneficial to human health and may be an adjunct therapy to increase the eradication rate of HP, lower treatment-associated side effects, and reduce HP-associated gastric inflammation. However, inconsistent test results have prevented conclusions about the therapeutic prowess of probiotics for HP. The mechanisms of actions of probiotics include the production of substances that inhibit or kill HP or compete with HP for the adhesion site on gastric epithelial cells. Probiotics can also reduce the release of inflammatory factors by regulating the local immune response of the host. We searched the available literature for full-length articles focusing on the role of probiotics in HP management. This review presents the latest advances in this area.

## 1. Introduction

*Helicobacter pylori* (HP) is the main cause of chronic active gastritis, peptic ulcer, gastric mucosa-associated lymphoid tissue lymphoma, and gastric cancer. Dyspepsia, unexplained anemia, and idiopathic thrombocytopenic purpura are also closely related [1]. Approximately half of all humans harbor HP which is the most common cause of chronic gastritis worldwide. The 2015 Kyoto consensus suggested that HP gastritis is an infectious disease and those who test positive should receive treatment designed to eradicate the infection [2]. At present, no therapy regimen can guarantee 100% eradication of HP. The eradication rate is related to many factors, including therapeutic regimen, patient tolerance to adverse reactions, patient compliance, patient genetic polymorphism, smoking, diabetes, and other factors [3–5]. Among them, antibiotic resistance of HP is the main cause of the failure of HP eradication treatment [6]. The Maastricht V/Florence consensus suggested that in areas where clarithromycin resistance exceeds 15% or in areas with high clarithromycin and metronidazole resistance, a 10–14-day bismuth quadruple therapy is recommended as the first-line eradication regimen [7]. In North America, the average rates of resistance of HP to metronidazole, clarithromycin, and levofloxacin between 2009 and 2011 were 20%, 16%, and 31%, respectively, of isolates [8]. A recent study from China reported average metronidazole, clarithromycin, and levofloxacin resistance rates of HP of 63.8%, 28.9%, and 28%, respectively, of isolates [9]. Although increasing the dose and course of antibiotics can increase the eradication rate of HP, there can be consequences. Severe adverse reactions during antibiotic therapy can include diarrhea, constipation, bloating, nausea, abdominal pain, abdominal discomfort, dysbacteriosis of the intestinal flora, liver function damage, and fungal infection. The reported rate of adverse reactions during the eradication therapy ranges from 5 to 30%, with treatment discontinued in some cases. Furthermore, the increased prevalence of *Escherichia coli*-resistant strains, methicillin-resistant *Staphylococcus aureus*, and extended-spectrum beta-lactamase strains isolated from the intestine after HP eradication treatment has been described [10]. In addition, bismuth is neurotoxic, which restricts the use in children and the elderly. In patients, gastric mucosa is acquired by gastroendoscopy for the culture of HP to determine antibiotic susceptibility. The prudent selection of antibiotics according to the results of drug susceptibility testing can effectively increase the eradication rate of HP. However, the harsh HP growth conditions and long growth cycle do not guarantee the success of HP culture, which has hindered the widespread use of HP culture techniques [11]. Molecular biology techniques, such as polymerase chain reaction (PCR) and fluorescent labeling nucleic acid in situ hybridization (FISH), can be used to detect HP resistance sites in fresh or paraffin-embedded gastric mucosa tissues and feces, but only to clarithromycin and quinolones. Metronidazole resistance sites cannot be determined due to the complex drug resistance mechanisms that are involved [12].

These challenges have spurred exploration of new individualized approaches to treat HP infections. The many adjuvant HP eradication treatments that have emerged include an oral HP vaccine [13], Chinese herbal medicine [14], probiotics and periodontal scaling [15, 16], and gastric mucosal protective agents [17]. Among them, probiotics have received increasing attention in recent years because of their safety. A large number of clinical and basic studies have reported that some specific probiotics can boost the HP eradication rate and significantly reduce the adverse reactions during the eradication treatment, which facilitates improved patient compliance with the therapy [18–20]. These attributes should seemingly make probiotics a promising adjuvant treatment.

However, according to the 2016 Toronto consensus, there is insufficient evidence that the addition of probiotics can increase the HP eradication rates and reduce adverse reactions [21]. The 2017 ACG clinical guideline, which was based on evidence from a meta-analysis, reported that probiotics can indeed increase HP eradication rates and reduce the overall incidence of adverse reactions. However, the studies involved in the meta-analysis were mainly clinical trials conducted in China and were subject to a high risk of bias. Thus, currently, there is no conclusion about the best choice of probiotics as well as the dose and course of treatment [8]. The fifth Chinese HP consensus opinion pointed out that the conclusion that some probiotic strains can alleviate gastrointestinal side effects following HP eradication is widely accepted. Whether the addition of probiotics can increase the HP eradication rate requires confirmation in future well-designed studies. For now, the anti-HP mechanisms of probiotics remain unclear. In the context of the high global prevalence of antibiotic resistance, determination of the roles of probiotics in the eradication of HP is important, as is the feasibility of using bacteria to cure bacteria. Here, we have a review of the latest advances in the role of probiotics in the treatment of HP infections.

## 2. Definition and Classification of Probiotics

The Food and Agriculture Organization of the United Nations and the World Health Organization define probiotics as living microorganisms that are beneficial to life; can tolerate the effects of stomach acid, bile, and pancreatic juice; can colonize the host's gastrointestinal tract or reproductive system; induce host reactions; and balance the intestinal flora to improve health [22]. Currently, compound active probiotics composed of various kinds of microorganisms are widely used globally, mainly for the treatment of diarrhea caused by dysbacteriosis of the intestine and to regulate the body's immune functions.

In 2013, the International Probiotics and Prebiotics Science Association classified probiotics as follows: (1) bacteria in the genus *Lactobacillus*, including *Lactobacillus acidophilus*, *Clostridium butyricum*, *L. reuteri*, *L. bulgaricus*, *L. casei*, *L. paracasei L. rhamnosus*, *L. salivarius*, and *L. plantarum*; (2) bacteria in the genus *Bifidobacterium*, including *Bifidobacterium infantis*, *B. adolescentis*, *B. animalis*, *B. longum*, *B. breve*, and ovary double *Bacteroides*; (3) Gram-positive cocci, such as *Streptococcus thermophilus*, *S. faecalis*, and *Lactococcus*; and (4) yeast, such as *Saccharomyces boulardii*. At present, commercially available probiotic products include probiotic amended yogurt, encapsulated live bacteria, bacteria powder, oral liquids, and various preparations of single strains.

## 3. Theoretical Basis of Microecological Therapy

Human skin and gastrointestinal, respiratory, and urogenital tracts harbor huge numbers of colonized microbes, which are important in regulating the immune function of the human body to resist the colonization of pathogens [23]. These microorganisms include beneficial bacteria, conditional pathogens, and pathogenic bacteria, which have evolved to a normal state of microecological balance in the human body. The gastric environment is particularly harsh and difficult for microbiota to colonize. The common wisdom for a long time was that the stomach was sterile for approximately 80% of microbes are not cultivable. With the development of high-throughput sequencing technology, this view has been debunked. HP is not the only inhabitant of the gastric mucosa anymore; a non-HP microbial community has been recognized and is called gastric microbiota [24]. HP may be influenced in their pathogenicity by the community they live in [25]. The gastric microbiota belong mainly to the Proteobacteria, Firmicutes, Actinobacteria, and Fusobacterium phyla, the majority of which were Streptococcus and Staphylococcus [26]. HP infection can affect the balance of gastric microbiota, and microbial interactions are a major factor in regulating the indigenous microbiota. Reports showed that the gastric microbiota of HP-negative subjects has a higher diversity than that of HP-positive patients [27].

Adhesion and virulence factors of HP contribute to pathogenicity. Colonization of the stomach by HP affects the distribution and quantity of the original gastric bacteria and upsets the microecological balance, resulting in disease. For example, there are fewer lactobacilli in the HP-infected stomach compared to the stomach not infected with HP [23]. HP leads to a microecological imbalance principally because of its production of an antibacterial peptide called cecropin. This peptide can cause other bacteria to undergo “autogenic autolysis” [28]. The lack of competition from these bacteria allows HP to multiply unimpeded. A series of virulence factors are able to stimulate the gastric epithelial cells, resulting in apoptosis and inflammation. In the context of the global prevalence of antibiotic resistance, increasing the dose of antibiotics or prolonging the course of antibiotics to increase the eradication rate of HP is not an ideal method, because it can promote the further development of antibiotic resistance.

Microecological therapy has brought new ideas to the treatment of HP. Remodeling the microecological balance in the stomach can reduce HP colonization. Concerning an animal model of HP infection, sterile, immunodeficient, or knockout animals are the easiest to establish. The use of ordinary mice is hampered by the difficulty to establish a chronic HP infection. An analysis of the components of the gastric microbiota in sterile and normal mice revealed that the number of bacteria in the stomach of normal mice reached was up to 10 × 8 colony forming units (CFU)/g, with Lactobacillus dominating [29]. In an animal experiment conducted in China, normal mice received HP suspensions for 7 days. The resulting HP infection rate was 30%. If the mice were first fed with a mixture of gentamicin and azithromycin for 3 days to eliminate the original microbiota of the stomach, the 7-day administration of HP produced a 100% HP infection rate. After the gastric microbiota balance was remodeled by feeding the Lactobacillus and Bifidobacterium suspension for 7 days, the HP infection rate was reduced to 30% and HP colonization decreased significantly. Some experiments confirm that some components of the gastric microbiota have been shown to exert antibacterial properties and could drive HP conversing from a spiral to a coccoidal form [30, 31]. The findings support the speculation that the immune system and normal gastric microbiota can effectively antagonize the colonization of HP, while disruption of the gastric microbiota balance increases the susceptibility to HP infection. The data provide a theoretical basis for the clinical use of probiotics to increase the HP eradication rate.

## 4. Effect of Probiotics on HP Eradication

Many meta-analyses and clinical trials have confirmed that probiotic supplementation can increase the eradication rate of HP and reduce adverse reactions during eradication. It can be concluded from literature analysis that not all probiotics have antagonistic effects on HP and different probiotics have specific effects. The antagonistic effect of mixed strains of probiotics on HP was greater than that of a single strain. Probiotics alone cannot completely eliminate HP but can reduce the amount of HP load in the stomach, reduce the delta value of UBT, and alleviate gastric mucosal inflammation. More details on the role of probiotics on the HP eradication rate can be seen in Tables 1–4.

Some clinical trials proved that probiotics can reduce the DOB values of UBT, despite a complete eradication of HP not being obtained [32–34]. Whether DOB values quantitatively reflect the density of gastric HP is a controversial question. DOB value is affected by many factors, such as the density of HP colonization, urease activity, and gastric emptying. Different probiotics can reduce the DOB value by inhibiting urease activity [35] or decreasing the attachment of HP to the gastric mucosa, suppressing the HP density [36].

## 5. Action Mechanism of Probiotics

### 5.1. Production of Substances That Inhibit or Kill HP

The antagonistic mechanism of probiotics to HP is unclear. Probiotic microorganisms can produce a variety of substances that inhibit HP and induce the secretion of antibodies by the host. A partial list of the antibacterial compounds includes bacteriocins, lactic acid, acetic acid, and hydrogen peroxide (H_2_O_2_). Different probiotic strains produce different antibacterial substances. *Strep. lactis* can produce nisin. This positively charged molecule can combine with cell membranes by electrostatic and hydrophobic interactions followed by membrane insertion to form a permeable channel that preludes cell autolysis and death [56]. *Bacillus subtilis* produces the antibiotic amicoumacin A and similar isocoumarins, and *L. roche* produces a variety of reoterms that all inhibit the growth and activity of HP. One of the characteristics of HP is the secretion of urease, which breaks down urea in the stomach to produce ammonia and which neutralizes gastric acid to protect the bacteria from gastric acid damage. Most lactobacilli produce lactic acid, which can inhibit the activity of urease. Lactic acid is deleterious to HP. The morphological alteration that occurs is independent of pH [57]. Fujimura et al. cocultured an HP standard strain and *L. gasseri* OLL2716 on agar for 24 h. Electron microscopy examination revealed a spherical shape of HP. The bacteria had also lost their growth ability [58]. Another characteristic of HP is catalase activity. Probiotics can produce H_2_O_2_. Catalase action results in the production of many oxygen radicals, which are antibacterial due to their interference with HP enzyme activity. Live probiotics antagonize HP, but bacterial viability may not be a prerequisite for the deleterious activity. Heat-inactivated *L. johnsonii* No. 1088 (HK-LJ88) can kill HP *in vitro* and, when cocultured with HP for 24 h, can lead to altered HP morphology and lysis. Orally administered HK-LJ88 can reduce HP colonization in the mouse stomach. The anti-HP effect of HK-LJ88 does not involve coagglutination of the bacteria. Rather, some surface molecules of HK-LJ88 are not inactivated by the heat [59]. In another study on the coculture of HP with *L. acidophilus* CRL 639 for 24 hours, the latter appeared to be lysed and the released protein compounds deformed or killed HP [60].

### 5.2. Effect on HP Colonization in the Stomach

The colonization of HP in the gastric epithelium is a prerequisite for the disease. HP has multiple flagella at one end, which provide the mechanical for a bacterium to penetrate the thick layer of the mucus and colonize the surface of gastric epithelial cells rather than being excreted with the peristalsis of the stomach. The HP surface contains adhesions, such as neutrophil activator protein, fibrillar N-acetylneuraminyl lactose-binding hemagglutinin (NLBH), Bab A, Lewis antigen, heat shock protein, Alp A, and Alp B. The gastric epithelial cells contain mucin receptors, mucopolysaccharide receptors, Lewis blood group substances, glycolipid receptors, and other corresponding receptors. The binding of the HP adhesins to the receptors mediates the colonization of HP in the gastric mucosa. Identifying the ability of probiotic bacteria to colonize the gastric mucosa is the first step in screening probiotics that can antagonize HP adhesion and colonization. Such probiotics can compete with HP for the adhesion site of gastric epithelial cells to reduce the colonization of HP in the stomach. Mukai et al. found that *L. reuteri* affects the colonization of HP by the secretion of sialic acid gangliosides and thiolates that inhibit HP's glycolipid linkage with gastric epithelial cells, as well as competing with HP for the adhesion of asialo-ganglio-N-tetraosylceramide and sulfatide [61]. *In vitro* experiments confirmed that Lactobacillus can downregulate the expression of HP adhesin sabA and reduce the adhesion of HP to gastric mucosa [62]. Others demonstrated that the growth of Lactobacillus and HP in the stomach is antagonistic to each other. If Lactobacilli exist in the stomach, the amount of HP will be reduced [63]. HP infection does not necessarily lead to disease, which is related to the virulence and quantity of HP. Peptic ulcers do not develop when the HP density of the gastric antrum is <10^5^ CFU/g [64]. The foregoing support the view that, while the oral administration of probiotics may well not completely eliminate HP, adhesion of HP can be reduced and gastric mucosal inflammation can be lessened. For children and elderly people who do not have digestive symptoms, oral administration of probiotics to reduce HP colonization is superior to traditional treatment.

### 5.3. Inhibition of Inflammation after HP Infection

HP infection leads to gastric mucosal inflammation. This can begin a pathway, which is termed the Correa cascade, from chronic gastritis to atrophic gastritis to intestinal metaplasia to atypical hyperplasia, culminating in gastric cancer. This is the most common pattern of evolution after gastric mucosal infection with HP. Urease, cytotoxin-associated gene A (CagA), vacuolating cytotoxin A (VacA), and neutrophil-activating protein (NAP) are common virulence factors of HP. As its name implies, NAP activates neutrophils. It also promotes the production of reactive oxygen species in neutrophils and mediates the adhesion of gastric epithelial cells to neutrophils, resulting the activation of gastric inflammation cascade reaction after HP infection. Interleukin- (IL-) 8 is the earliest discovered cytokine associated with HP gastritis. It is a chemotactic compound that can activate neutrophils, induce the degeneration and necrosis of gastric mucosa cells, and also stimulate mucosal monocytes and dendritic cells to produce tumor necrosis factor, IL-1, and IL-6. The dendritic cell activity produces an inflammatory cascade. These responses are insufficient to clear HP infection but cause chronic inflammation [65]. Virulence and inflammatory factors are now being used to prepare HP-associated vaccines. Animal studies have demonstrated the considerable (80%) effectiveness of oral vaccines based on NAP [66]. Some probiotic strains can also reduce the release of inflammatory factors by regulating the local immune response of the host and relieving the inflammatory response of the gastric mucosa. An *in vitro* study confirmed that HP-induced secretion of IL-8 in gastric epithelial cells can be reduced by *L. salivarius* [67]. Another study showed that the exopolysaccharide of *Strep. thermophilus* CRL1190 reduces the colonization of HP to AGS cells and also relieves the inflammatory response of AGS cells caused by HP [68]. The mechanism by which probiotics regulate mucosal immune responses is unclear. *L. reuteri* can inhibit the activation of nuclear factor-kappa B (NF-*κ*B) and downstream factors by blocking the release of the tumor necrosis factor from macrophages. *L. acidophilus* can inhibit the expression of Smad7, inactivate the transduction of the NF-*κ*B pathway, and weaken the HP-induced gastric mucosal inflammatory response [69]. *L. plantarum* and *L. acidophilus* applied prior to HP infection reduce the degree of gastritis. Phosphorylation of Janus kinase 2 and the expression of the cytostatic factor suppressors of cytokine signaling- (SOCS-) 2 and 3 are increased in the JAK-STAT pathway. SOCS-2 and SOCS-3 can inhibit a variety of signal transduction pathways, which reduces the release of inflammatory factors [70]. Probiotics can reduce the release of IL-8, interferon gamma, and other inflammatory factors by inhibiting the Toll-like receptor 4-NF-*κ*B signaling pathway [71]. *L. salivarius* UCC118 and UCC119 can reduce the secretion of IL-8 by the gastric mucosa after HP infection. This effect has nothing to do with the life or death of *L. salivarius*, but the probiotic body must be complete. *L. salivarius* UCC118 and UCC119 can destroy the type IV secretion system encoded by the cagPAI of the HP toxin-related gene and block the entry of the effector molecule cagA into host epithelial cells [72]. A probiotic mixture of *Enterococcus faecalis*, *B. longum*, and *L. acidophilus* reportedly tolerated the acidic environment in the stomach and survived for 8 hours. These three probiotics could not reduce the colonization of HP in the stomach but could reduce the release of inflammatory factors such as tumor necrosis factor-alpha, IL-1*β*, IL-10, IL-6, granulocyte colony-stimulating factor, and macrophage inflammatory protein 2 by inhibiting the NF-*κ*B and mitogen-activated protein kinase signal transduction pathways [73]. Arginine is a substrate for the synthesis of nitric oxide, one of the strongest mediators of inflammation. The arginine deiminase activity following L. brevis administration causes arginine deficiency and prevents polyamine generation from proliferating cells [52].

## 6. Conclusion

With the deepening of the research on the intestinal microflora, microecological therapy is attracting increasing attention. Probiotics can help improve the eradication rate of HP and reduce the adverse reactions. However, not all probiotics but only some specific probiotic strains have such effects. Here are several research hurdles still to be surmounted. First, the gut microflora of humans is affected by various factors, such as the environment, diet, genetics, and lifestyle, and it is difficult to directly study the effects of probiotics on the human body. Second, due to the synergistic or antagonistic effect between bacteria, it is difficult to generalize the effects of certain probiotic strains in the different probiotic combinations. Third, due to the specificity of the strains and the inconsistent results of the research, the results to date can be questioned.

The results to date consistently support the prowess of probiotics in alleviating adverse reactions in the eradication of HP. However, questions remain. Can probiotics increase the eradication rate of HP? If so, what is the mechanism? Which probiotic strain has the best anti-HP effect? What is the best dose and the timing of medication (i.e., before or after eradication)? Is there a difference in efficacy between single strains and mixed strains? Are there side effects of supplemental exogenous probiotics? The answers await future basic and clinical studies.

## Figures and Tables

**Table 1:** Summary of meta-analysis of the effect of probiotics on the eradication rate of HP.

Author	Trials	Probiotic	Result
Jian et al. [37]	8 RCT (*n* = 1372)	Lactobacilli + triple therapy	Pooled eradication rate Probiotic: 82.26% (95% CI = 78.01–86.51%) No probiotic: 76.97% (95% CI = 73.11–80.83%) OR = 1.78 (95% CI = 1.21–2.62)
Sachdeva and Nagpal [38]	10 RCT (*n* = 963)	Multistrain (fermented milk) + triple or quadruple therapy	Eradication rates were improved by approximately 5–15% OR = 1.91 (95% CI: 1.38–2.67)
Dang et al.[39]	33 RCT (*n* = 4459), 9 RCT for children, 24 RCT for adults	Probiotics + triple therapy or sequential therapy or quadruple therapy	The pooled eradication rate in probiotic supplementation groups was significantly higher than that in controls (RR = 1.122, 95% CI = 1.086–1.159)
Szajewska et al.[40]	9 RCT (adult, *n* = 1708) 2 RCT (children, *n* = 330)	Saccharomyces boulardii + triple therapy	Eradication rate (adult) Probiotic: 80.0% (95% CI = 77–82) No probiotic: 71.0% (95% CI = 68–74) RR = 1.11, 95% CI = 1.06–1.17 Eradication rate (children) Probiotic: 87.5% No probiotic:77.2% RR = 1.13, 95% CI = 1.03–1.25
Zhang et al. [41]	45 RCT (*n* = 6997)	Probiotics + standard therapy	Eradication rate Probiotic: 82.31% No probiotics: 72.08% (RR: 1.11; 95% CI: 1.08–1.15) Side effects (RR = 0.59; 95% CI = 0.48–0.71) Probiotic: 21.44% No probiotic: 36.27%
Wen et al. [42]	17 RCT in Asian pediatric patients (*n* = 1932)	Multistrain probiotics + 14-day triple therapy	Bifidobacterium infantis + Clostridium butyricum was most beneficial for eradication rates (RR: 1.16, 95% CI: 1.07–1.26)
Losurdo et al. [43]	7 RCT (*n* = 517)	Probiotic strain alone	The mean weighted eradication rate was 14% (95% CI = 2%–25%) Lactobacilli: 16% (95% CI: 1%–31%) Saccharomyces boulardii: 12% (95% CI: 0%–29%) Multistrain: 14% (95% CI: 0%–43%)

**Table 2:** Summary of clinical trials using a single strain of probiotics with antibiotics.

Author	Study size	Probiotic	Study type	Result
Zhao et al. [44]	240	Saccharomyces boulardii	A prospective, randomized, controlled study	Eradication rate Probiotic: 85.0% No probiotic: 75.8% Adverse reaction decreases
Dore et al. [45]	45	Lactobacillus reuteri (DSM 17938)	A case report series	Eradication rate: 93.3%
Cekin et al. [18]	159	Bifidobacterium animalis subsp. lactis B94	Randomized, placebo-controlled study	Eradication rate Probiotic: 86.8% No probiotics: 70.8% Adverse reaction decreases
Zhu et al. [46]	240	Saccharomyces boulardii	Randomized clinical trial	Saccharomyces boulardii reduced the overall side effect rate, and there was no difference observed in efficacy on the eradication rate
Chen et al. [47]	105	Clostridium butyricum	Open-label, randomized clinical trial	No significant difference in eradication rates was observed. Supplementation of probiotics led to improvement of gastrointestinal symptoms

**Table 3:** Summary of clinical trials using mixtures of probiotics in association with triple therapy.

Author	Study size	Probiotics	Study type	Result
Du et al. [48]	228	L. acidophilus + S. faecalis + B. subtilis	Randomized Prospective Open	Eradication rate Probiotic: 79.5% No probiotics: 60.8% Adverse reaction decreases
Wang and Huang [49]	100	L. acidophilus + B. bifidum	Randomized Prospective Open	Probiotic: 83.7% No probiotics: 64.4%
Tongtawee et al. [50]	200	Lactobacillus delbrueckii + Streptococcus thermophillus	Double-blind Placebo-controlled Randomized	Probiotic: 90.8% No probiotics: 84.3%
Haghdoost et al. [51]	176	Lactobacillus + Bifidobacterium	Randomized Placebo-controlled study	Probiotic: 78.4% No probiotics: 64.8%

**Table 4:** Summary of clinical trials using probiotics in the absence of antibiotics.

Author	Study size	Probiotic	Study type	Result
Sakamoto et al. [33]	31	Lactobacillus gasseri OLL2716	A randomized, controlled clinical trial	Value of UBT decreases. Examination of antral biopsies showed two- to 100-fold decreases in the numbers of HP, but in no case were bacteria eliminated completely.
Cruchet et al. [34]	326	Lactobacillus johnsonii La1	A double-blind, randomized, controlled clinical trial	A significant difference (DOB2–DOB1) was detected (−7.64 per thousand; 95% CI: −14.23 to −1.03)
Linsalata et al. [52]	22	Lactobacillus brevis	A randomized, double-blind, placebo-controlled study	Reduction in the UBT delta values
Imase et al. [36]	179	L. reuteri	A randomized, double-blind, crossover study	Administration of L. reuteri tablets significantly decreased UBT in HP-positive subjects
Rosania et al. [53]	80	A mixture of 8 different probiotics	Double-blind, placebo-controlled, randomized	Eradication rate Probiotic: 32.5% Placebo: 0%
Francavilla et al. [54]	100	L. reuteri DSM 17938 + L. reuteri ATCC PTA 6475	Double-blind, placebo-controlled, randomized	Probiotic: a decrease in the 13C-UBT value by 13% Placebo: a 4% increase
Holz et al. [55]	128 subjects, 47 twin pairs, and 34 singletons	L. reuteri DSM 17648	Placebo-controlled pilot study	Had unique properties as it specifically aggregates with planktonic HP in the stomach. It can significantly reduce the HP load after a 14-day oral treatment
